# From theory to RAPID AMOC observations: a personal voyage of discovery

**DOI:** 10.1098/rsta.2022.0192

**Published:** 2023-12-11

**Authors:** Jochem Marotzke

**Affiliations:** ^1^ Max-Planck-Institut für Meteorologie, Bundesstrasse 53, 20146 Hamburg, Germany; ^2^ Centrum für Erdsystemforschung und Nachhaltigkeit, Universität Hamburg, Hamburg, Germany

**Keywords:** Atlantic Meridional Overturning Circulation, circulation monitoring, AMOC theory

## Abstract

I review the history of ideas that have led to the establishment of the RAPID monitoring system for the Atlantic Meridional Overturning Circulation (AMOC) at 26.5° N. This history is closely connected to important events in my personal career. Starting from early and largely unsuccessful attempts at formulating a dynamically consistent force balance for the AMOC, I made theoretical progress by separately predicting the density at the eastern and western boundaries and by invoking exact geostrophic balance throughout, including the western boundary current. A remarkable confluence of individuals and ideas then enabled the establishment of the RAPID array, at its core based on monitoring boundary densities and on geostrophy. The RAPID results, such as the surprisingly large sub-seasonal variability, have encouraged AMOC monitoring approaches at other latitudes. I finish by pointing at two theoretical concepts—first, acknowledging the difference between convective mixing and sinking and, second, considering the advective rather than wave propagation of density perturbations in the deep western boundary current—that, together with continued observations and newly available global coupled simulations at very high resolution, should substantially improve our understanding of the causes of AMOC variability.

This article is part of a discussion meeting issue 'Atlantic overturning: new observations and challenges'.

## 1. Introduction

The RAPID array has been monitoring the Atlantic Meridional Overturning Circulation (AMOC) at 26.5° N since April 2004. This observing system has for the first time provided a continuous estimate of one of the climatically most important dynamical quantities in the ocean. The early papers based on these measurements confirmed the monitoring design [1,2], showed surprisingly large sub-seasonal fluctuations [3], quantified the AMOC seasonal cycle [4,5] and provided the first continuous estimate of meridional heat transport in the ocean [6]. The data have now been analysed up to December 2020 and have been used in hundreds of publications [7]. It appears timely to review the history of ideas that formed the foundation of the RAPID monitoring system, as well as to present some consideration of the RAPID impact.

I should state at the outset that this review cannot be a proper historical account. Such an endeavour would need to be undertaken by a professional historian of science who is unrelated to RAPID. However, the development of the ideas leading to the RAPID system started almost 40 years ago and has been closely connected to important moments in my personal career. Hence, I take the liberty offered to me by this Royal Society Discussion Meeting, which was meant by the organizers also to cover the origins of the RAPID system, to weave together personal and scientific elements. The result is reminiscent of the literary genre of ‘narrative nonfiction’, which has already been applied to physical oceanography and the AMOC in the book by Dallas Murphy [8]. By contrast, the current paper aims at a professional audience and develops in depth the physical-oceanography foundations of AMOC monitoring.

## 2. An unfinished part of a PhD thesis

A few days after the start of my PhD work by mid-1985, my advisor Jürgen Willebrand introduced me to a distinguished visitor to the Institut für Meereskunde in Kiel: Pierre Welander had brought with him the idea for a research project aiming at the stability and multiple equilibria of the ocean's thermohaline circulation (THC). The motivation arose from the groundbreaking paper by Bryan [9], of which we had a draft version, and which showed for the first time with an ocean general circulation model (OGCM) that an idealized single-basin, pole-to-pole configuration could have at least two fundamentally different equilibria under the same equatorially symmetric surface forcing. One equilibrium showed a THC symmetric about the equator, with sinking in both high latitudes; the other equilibrium showed a single pole-to-pole THC cell.

At that time the running of OGCMs over the long THC timescales was considered too cumbersome for many research groups. Therefore, Pierre suggested to me the equations for a simplified model of the THC. This model was supposed to capture the essential elements but should be two orders of magnitude more computationally efficient than an OGCM. The model was formulated in a latitude-depth plane and comprised, in addition to temperature and salinity conservation, the momentum equations in their geostrophic approximation plus vertical friction. Upon zonal averaging, we obtained

2.1 $$-f\bar{v}=-\frac{p_{E}-p_{W}}{\rho_{{\;}_{0}}L_{x}}+A\partial_{zz}\bar{u}$$

and

2.2 $$f\bar{u}=-\frac{1}{\rho_{{\;}_{0}}}\partial_{y}\bar{p}+A\partial_{zz}\bar{v},$$

where *u* and *v* are the zonal and meridional velocities, respectively, the overbar marks the zonal average, f is the Coriolis parameter, *L*_x_ is the zonal width of the basin, *p*_E_ and *p*_W_ are the pressures at the eastern and western boundaries, respectively, *ρ*_0_ is a reference density and *A* is the vertical friction coefficient.

The problem arising when we wanted to construct a zonally averaged model from (2.1) and (2.2) was obvious: We should only have barred quantities, and the boundary-pressure terms in (2.1) prevented this closure. And since geostrophy implied that the dominant balances were between the left-hand sides and the first terms on the right-hand-sides of (2.1) and (2.2), the problem was not ever going to disappear. How would one then proceed? Pierre Welander's suggestion was one of blatant assertion—to ignore (2.1) altogether and to ignore the large term on the left-hand-side of (2.2). Such a procedure could be rationalized by assuming a very sticky substance—honey rather than water—and thus assuming large vertical friction. Alternatively, one could assume a non-rotating system. The resulting dynamical equation,

2.3 $$\frac{1}{\rho_{{\;}_{0}}}\partial_{y}\bar{p}=A\partial_{zz}\bar{v},$$

allowed us to compute the zonally averaged meridional velocity from the zonally averaged meridional pressure gradient and thus provided the desired closure.

We published a paper with the two-dimensional THC model based on (2.3) [10], but none of us were satisfied with the justification for the dynamical balance. When Pierre Welander returned to Kiel in 1986, he asked me whether I had made progress on the closure problem. I said: ‘No, but I tried’. He said: ‘Try again’. I did, but without any success. I eventually sidestepped the closure problem by using a three-dimensional OGCM and managed to finish my thesis [11] without solving the closure problem. Apart from my first two papers [10,12] and my thesis, I never again used the two-dimensional THC model in any published work. But the closure problem of the three-dimensional THC has remained a bit of an obsession for me ever since.

## 3. Theoretical advances in the 1990s

Around 1990, Thomas Stocker and Dan Wright constructed a two-dimensional THC model [13] that was much more sophisticated than ours. In particular, they approached the closure problem by converting the momentum equations into a vorticity equation, thereby eliminating the geostrophic balance, and they developed parameterizations for the zonally averaged vorticity balance (see [14,15] for comprehensive analyses). Their approach crucially relied on dissipation of relative vorticity in frictional boundary layers, thus emphasizing ageostrophic effects. Their model showed some impressive successes when compared with the results of OGCMs, in relating meridional flow strength to meridional density differences (e.g. fig. 3*b* in [15]).

Despite these successes, I felt that the analysis in Wright *et al*. [14] and Wright *et al*. [15] left a fundamental gap. To the best of my understanding, they never returned to (2.1), the zonally averaged equation for the zonal momentum balance. But there was, and still is, the observational fact that all large-scale oceanic flow below the Ekman layer is in geostrophic balance to an accuracy beyond observational capabilities (e.g. [16,17]); furthermore, by the observational knowledge of the 1990s, all analyses of hydrographic sections gave a stable result of northward integrated transport above 1000 m depth in the subtropical North Atlantic (e.g. [18,19]). Hence there ought to be a robust relationship between the pressures (or densities) at the eastern and western boundaries of the North Atlantic. A strong northward THC should be reflected in a definitive relationship between the pressure terms in (2.1), and any satisfactory dynamical closure for the THC should speak to this relationship. By contrast, the approach by Wright, Stocker and colleagues stressed ageostrophic effects.

**Figure 1.**
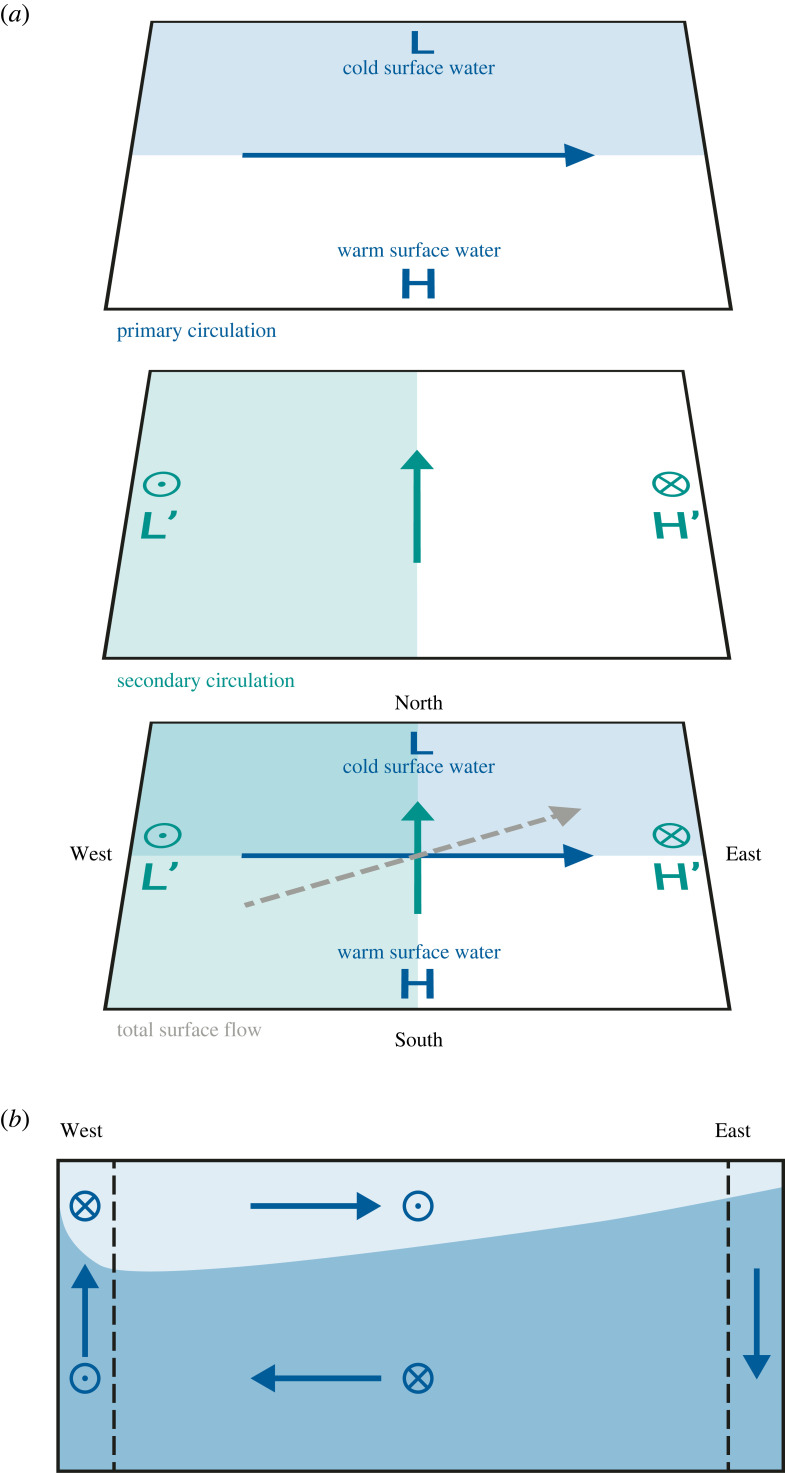
Conceptual picture of the THC after Zhang *et al*. [20] and Colin de Verdière [21]. (*a*) Plane view; H and L mark the primary high and low sea level, respectively, and the blue arrow marks the primary, eastward geostrophic surface flow. Stylized arrow heads and tails mark the resulting upwelling and downwelling, respectively, which cause the secondary low L’ and secondary high H’, respectively. The turquoise arrow marks the secondary circulation, the zonally integrated surface flow. The grey dashed arrow indicates the total surface flow, from the south-west to the north-east. (*b*) Zonal section representing the conceptual picture, with eastward surface flow and westward abyssal flow, upwelling at the western boundary and downwelling at the eastern boundary. Note that this representation implies thermocline and deep subtropical gyres, with surface and deep western boundary currents and the thermocline sloping upward toward the east, implying southward thermocline flow. Stylized arrow heads and tails mark southward and northward flow, respectively. Panel (*b*) was redrawn after Colin de Verdière [21].

While the success of the Stocker and Wright model—and by implication the success of the vorticity closure approach—might have put the question of geostrophic balance of the THC onto the back burner, a few theoreticians pushed ahead, at least on qualitative terms. Simultaneously and independently, Zhang *et al*. [20] and Colin de Verdière [21] put forward similar conceptual pictures for the force balance of the THC (figure 1). If we assume that the surface ocean has low density at low latitudes and high density at high latitudes, a simple two-layer picture implies high sea level at low latitudes and low sea level at high latitudes. Resulting is an eastward geostrophic surface current, which impinges on the eastern boundary layer, causes a pile-up (high sea level) there, and sinks down. The eastward surface current is fed at the western boundary layer, implying upwelling there. The pattern of upwelling at the western and downwelling at the eastern boundary would cause relatively high thermocline density at the western boundary and relatively low thermocline density at the eastern boundary. This would create a secondary low in sea level at the western boundary and a secondary high in sea level at the eastern boundary; geostrophic balance integrated zonally would create an integrated near-surface transport to the north as a secondary circulation. The overall surface circulation would go from the south-west to the north-east.

Appealing as the conceptual picture of Zhang *et al*. [20] and Colin de Verdière [21] was, neither paper offered any quantification of their concept of a three-dimensional THC. Within these two papers, this made perfect sense, because the conceptual picture was meant to give a qualitative explanation for the three-dimensional flow structures found in numerical models. I made some preliminary attempts to cast the conceptual picture into quantitative shape and to find numerical solutions. However, my attempts were met with utter failure; for instance, the THC was maximum at the southern, rather than near northern boundary, because of the smaller f near the southern boundary. Furthermore, the THC became stronger instead of weaker with weaker mixing; the reason was that without additional processes to ensure connectivity along the side boundaries and the equator (see below), a diffusive boundary layer formed near the bottom of the eastern boundary. Smaller diffusion meant a narrower boundary layer and stronger downward penetration of low surface density, in turn providing stronger thermal wind shear. I could see no way out of this quandary, and I left the problem aside.

The fact that neither Zhang *et al*. [20] nor Colin de Verdière [21] provided a closed-form solution for the THC as a function of the independent parameters, or at least an order-of-magnitude estimate from scaling assumptions that was based on the conceptual picture, meant that we were still without a theoretical estimate of THC strength that showed at least rudimentary dynamical consistency. Hence, the only extant theoretical estimate of THC strength was one that had taken its starting point in the thermocline scaling of Welander [22] and was later applied to the THC by Bryan [23]. But this scaling had serious inconsistencies, as already noted by Bryan [23] and repeated now.

The scaling was constructed as follows. If we assume that the atmosphere imposes onto the ocean a zonally symmetric surface distribution of density, thermal wind suggests a scaling for the zonal flow according to

3.1 $$f\partial_{z}u=\frac{g}{\rho_{{\;}_{0}}}\partial_{y}\rho \;\Rightarrow \;f\frac{U}{D}=\frac{g}{\rho_{{\;}_{0}}}\frac{\Delta \rho }{L},$$

where g is the gravitational constant and *ρ* the *in-situ* density; U, *Δρ*, *D* and *L* are scales for zonal flow, the surface density contrast between high and low latitudes, and vertical and meridional extent, respectively.

The next ingredient is vertical advective-diffusive balance, an assumption that is standard in physical oceanography (e.g. [24]), although its neglect of horizontal transport terms is rightly viewed with suspicion. Let us still proceed to obtain the scaling

3.2 $$w\partial_{z}\rho =k_{v}\partial_{zz}\rho \;\Rightarrow \;\frac{W\Delta \rho }{D}=\frac{k_{v}\Delta \rho }{D^{2}}\;\Rightarrow \;W=\frac{k_{v}}{D},$$

where *w* is vertical velocity, *k*_*v*_ the vertical diffusivity and *W* the vertical velocity scale.

The last ingredient of the simple scaling is the continuity equation, which under the Boussinesq approximation takes the form of the incompressibility condition. Upon zonal averaging we obtain

3.3 $$\partial_{x}u+\partial_{y}v+\partial_{z}w=0\Rightarrow \partial_{y}\bar{v}+\partial_{z}\bar{w}=0\;\Rightarrow \;\frac{U}{L}=\frac{W}{D},$$

where we have used the same scaling for v as for u, a point we will return to. Equations (3.1)–(3.3) give three conditions for the three scaling unknowns *U*, *W* and *D*. Once solved for, these can be combined into a volume transport or meridional overturning stream function *ψ* by assuming a flow through a cross section, which gives the scaling solution for *ψ*,

3.4 $$\psi =UDL=WL^{2}={\left(\frac{g\Delta \rho L^{4}k_{v}^{2}}{f\rho_{{\;}_{0}}}\right)}^{1/3}.$$

Using g = 10 m s^−2^, Δ*ρ* = 4 kg m^−3^, L = 5 × 10^6^ m, k_v_ = 10^−4^ m^2^ s^−1^, f = 10^−4^ s^−1^ and *ρ*_0_ =10^3^ kg m^−3^, we obtain the very reasonable estimate for the maximum meridional overturning

3.5 $$\psi \approx 2.5^{\;1/3}\times 10^{7}\;{\text{m}}^{\text{3}}\;{\text{s}}^{-1}\approx 13.6\;\text{Sv, }$$

with 1 Sverdrup (Sv) ≡ 10^6^ m^3^ s^−1^.

What was wrong with the scaling solution (3.4)? Note that the two-thirds power dependence of *ψ* on *k*_v_ has been found empirically in many simulations (e.g. [25,26]), so my criticism aims at the method of derivation rather than at the result itself. As already noted by Bryan [23], we took quite some liberty in using the scaling for the *zonal* flow in the scaling for the *meridional* flow. But zonally averaged meridional flow is potentially a small residual of western boundary current and return flow in the eastern basin, as the meridional overturning circulation (MOC) in the North Pacific shows (e.g. [27,28]), so there is no *a priori* reason why isotropy—$\bar{u}$ and $\bar{v}$ scale alike—should be a good assumption. Instead, a scaling for the zonal-mean *meridional* flow should invoke the zonal-mean zonal pressure gradient—the pressure difference between eastern and western boundaries—just as it showed up in the closure problem (2.1) of the two-dimensional models! Perhaps because this problem had become the above-mentioned obsession for me, I was struck by the fact that paper upon paper repeated the scaling (3.4) without being bothered by the apparent violation of geostrophy and thus Newton's second law. A close colleague once characterized this gap in reasoning as minor. I felt, however, that at a minimum we ought to make explicit the working hypothesis of the zonal pressure gradient scaling linearly with the meridional pressure gradient, a point clearly acknowledged by Bryan [23] but not by many followers.

The incorporation of thermal wind was not the only problem in the scaling (3.4). As pointed out much later by Jeff Scott in his thesis at MIT [29], the vertical scale *D* appears in (3.1)–(3.4) in three different meanings: In the thermal-wind equation (3.1), *D* denotes the vertical range of substantial baroclinicity. In the vertical advective-diffusive balance (3.2), *D* denotes the depth over which surface density approaches abyssal density up to a relative residual of 1/*e*. In the continuity equation (3.3), finally, *D* denotes the depth of the maximum overturning or, loosely, the level-of-no-motion. There is no reason that the three definitions of *D* should be equivalent or should even scale similarly, and indeed Scott [29] found different power laws for the dependence of the three definitions of *D* on *k*_*v*_.

The state of theoretical understanding of the THC in the mid-1990s was eloquently characterized by Alain Colin de Verdière in his review of Joseph Pedlosky's book ‘Ocean Circulation Theory’ ([30], p. 98, the emphasis is mine): ‘The thermohaline circulation problem, on the other hand, requires the parallel computation of both density and velocity fields and is only briefly touched upon [in Pedlosky's book]. Most recent advances on the latter topic motivated by the explosive interest in climate have come from numerical simulations and *there are still many steps to be ascended on the stairway linking these numerical results and first principles*’.

## 4. Boundary mixing and the dynamics of three-dimensional thermohaline circulations

**Figure 2.**
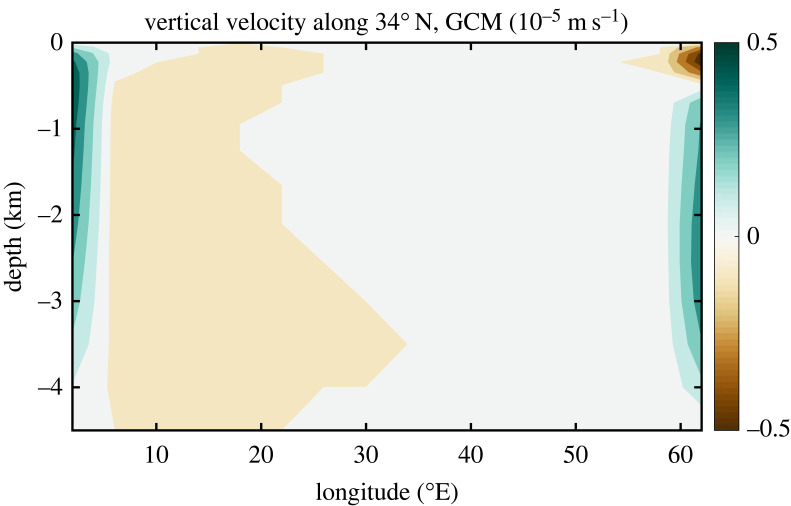
Vertical velocity along 34° N in the standard numerical solution of Marotzke [25].

Nothing much was happening in the area of THC theory when one day in 1995, during one of our frequent drives from the Massachusetts Institute of Technology (MIT) to the Woods Hole Oceanographic Institution, Carl Wunsch asked me in his inimitable style: ‘Why do modellers keep ignoring the observational evidence of enhanced mixing near ocean boundaries?’ My truthful answer was that (a) I did not really know, that (b) the standard answer referred to numerical instability ensuing if interior mixing was very weak or absent [31], and that (c) I was not sure anyone had actually tried but that I would give it a shot. Resulting was a paper [25] with the first numerical experiments that had vertical mixing only at the side boundaries and in the convective areas. The numerical solution showed—among other interesting results such that the solution was indeed numerically stable—vertical velocities entirely confined to the side boundaries, that is, in the regions of vertical mixing (figure 2; note that there is no wind forcing in this simulation). This stood in marked contrast to the expectations from Stommel–Arons theory (e.g. [32]), which had upwelling either uniform or varying over planetary scales only. But this also stood in marked contrast to the expectation from figure 1*b* [21]: At the western boundary, we see the expected upwelling. But at the eastern boundary, we see a pattern of downwelling above upwelling, with the transition depth deepening with increasing latitude (not shown), and implying a three-layer zonal flow – eastward flow at the surface and in the abyss, and westward flow in between (the mass balance at the eastern boundary is purely between vertical and zonal flows).

**Figure 3.**
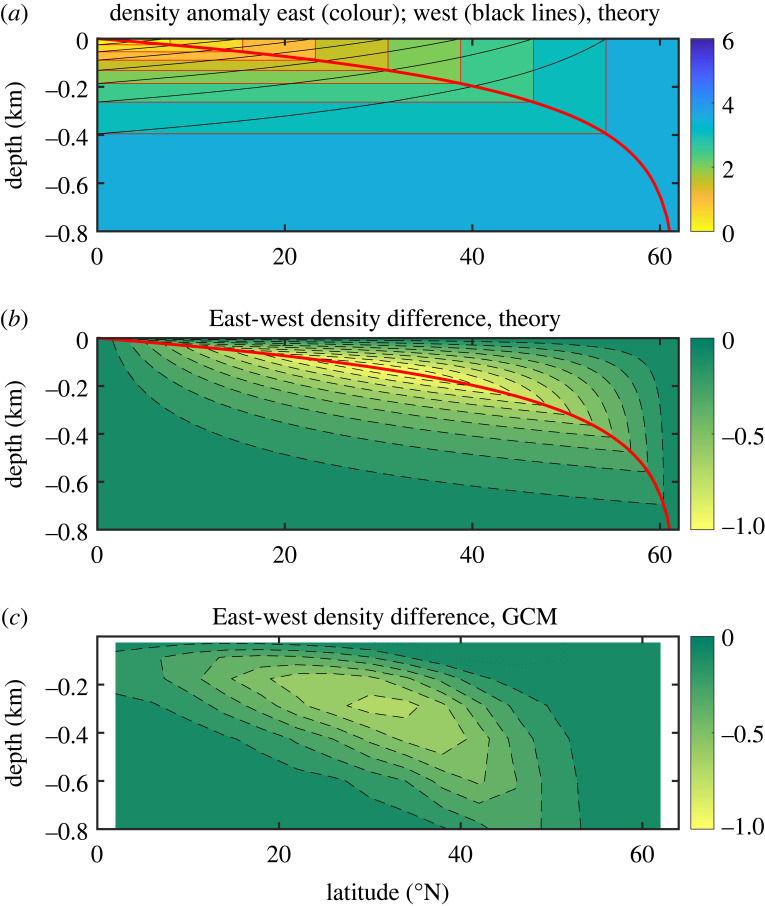
(*a*) ‘Stacked boxes’ underlying the THC theory; shown is the density anomaly (in kg m^−3^), relative to the equatorial surface, for the eastern and western boundaries. (*b*) Density difference (in kg m^−3^) between eastern and western boundaries, based on the stacked boxes. (*c*) Potential density difference (in kg m^−3^) between eastern and western boundaries, from the GCM. The thick red line marks the depth of convection; dashed contours indicate negative values. Only the top 800 m of ocean are shown. After Marotzke [25].

The clear relationship between vertical velocity and the presence of vertical mixing encouraged me to return to the attempts at finding closure for the force balance of the THC. In contrast to the previous closures in two-dimensional models, which all tried to express the model solely in terms of zonal averages, I focused on determining density in the eastern and western boundaries separately. I took as my starting point the conceptual picture of figure 1 but formulated two additional crucial ingredients (assumptions (iv) and (v) below). Overall, I started from five fundamental assumptions, in addition to the standard hydrostatic and geostrophic approximations:

(i) Surface density is given and is a function of latitude only; the abyss uniformly has the properties of the densest surface water.
(ii) The western boundary water is assumed to be stably stratified, following an exponential with scale height *D* (to be determined as part of the solution).
(iii) Density in the lateral boundary layers is governed by vertical advective-diffusive balance, except where convective mixing is present, which then also enters the balance.
(iv) Since there is no wind stress in this model, no zonal pressure gradient can be supported at the equator, and isopycnals are level along the equator.
(v) Along the eastern boundary, convective mixing occurs down to a depth *z_ρ_*, which is a function of latitude and which is determined as part of the solution. Hence, the isopycnal that outcrops at any given latitude is vertical down to depth *z_ρ_*. Equatorward, this isopycnal is assumed level.

Assumption (i) was used before by Welander [22] and Bryan [23]; (ii) and (iii) are standard assumptions and underlie the Bryan [23] scaling and indirectly the Stommel–Arons picture. Here, vertical advective-diffusive balance is more likely to apply since near the boundaries, both vertical mixing and vertical motion are strong. Assumption (iv) stems from the force balance between wind stress and pressure gradient, which is traditionally assumed in equatorial oceanography; a pressure gradient implies a thermocline slope. Assumption (v) is the most unorthodox; it is based on the physical picture that warm water generally moves to the northeast (figure 1), so subsurface advection of a certain density can occur only until the outcrop latitude of this isopycnal is reached. That the isopycnals should be level equatorward of the outcrop latitude could be caused by Kelvin waves, which were later invoked by Johnson & Marshall [33] in what could be viewed as indirect justification of the assumption of level isopycnals here. This assumption is, however, neither strictly required nor confirmed by the numerical experiments of Marotzke [25]. Indeed, the three-level structure in zonal flow implied by figure 2 requires some sloping-down of isopycnals as they approach their outcrop latitude. Nevertheless, we assume level isopycnals equatorward of the outcropping latitude for analytical convenience. Note that level isopycnals at the eastern boundary were also assumed in the famous ventilated-thermocline theory of Luyten *et al*. [34], a fact overlooked in Marotzke [25].

Our assumptions result in a picture of the density structure resembling ‘stacked boxes’, a term coined by Jeff Scott (figure 3). The stacked boxes allow us to calculate the meridional overturning circulation, once the basic stratification parameter—thermocline depth *D*—is known. The logic is as follows. Assume that *D* is known. This implies that the density is known all along the western boundary. In particular, the depth of any isopycnal at the western boundary is known at the equator. It is known for all longitudes at the equator because the isopycnal slope is zero. This depth *z_ρ_* is also known along the eastern boundary, all the way northward to the outcrop latitude. Hence, density along the eastern wall is known, and we can calculate the east-west density difference.

This difference allows us to compute the meridional overturning as follows. Geostrophic balance for the zonal-mean meridional velocity gives, starting from (2.1),

4.1 $$f\bar{v}=\frac{p_{E}-p_{W}}{\rho_{{\;}_{0}}L_{x}}.$$

Assuming vertical side boundaries allows us to take the vertical derivative of (4.1) to obtain

4.2 $$f\partial_{z}\bar{v}=-g\frac{\rho_{E}-\rho_{W}}{\rho_{{\;}_{0}}L_{x}},$$

where we have used the hydrostatic relationship. The meridional overturning stream function, which can be defined because of the two-dimensional continuity equation (3.3), then relates to the east-west density difference according to

4.3 $$\partial_{zz}\psi =-\partial_{z}\bar{v}=g\frac{\rho_{E}-\rho_{W}}{f\rho_{{\;}_{0}}L_{x}}.$$

**Figure 4.**
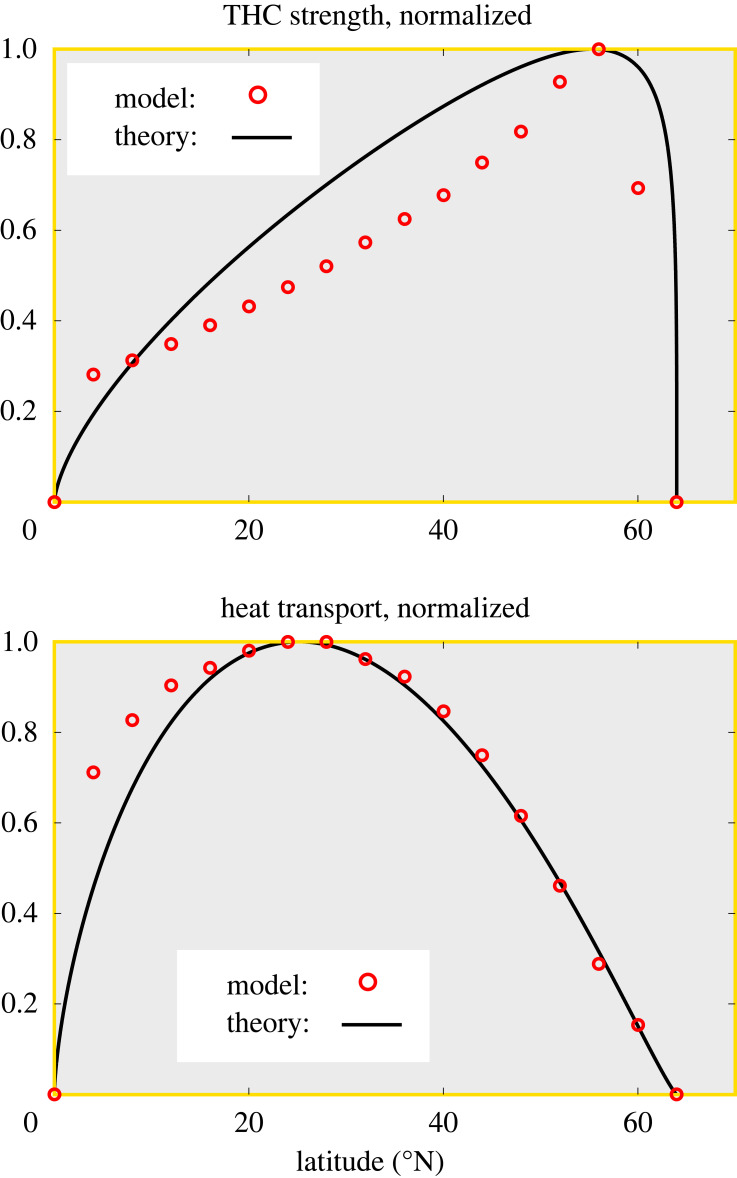
Latitudinal dependence of THC strength (top) and heat transport (bottom), according to the theory (solid line) and from the GCM (maximum THC strength and heat transport at every latitudinal grid point, red circles). Note that theory and GCM results have been scaled separately each to its maximum. After Marotzke [25].

Equation (4.3) allows us to calculate the overturning from the east-west density difference by twice integrating vertically and assuming a sensible reference level (not a trivial assumption). Overall, this logical sequence allows us to calculate the overturning, including $\bar{w}$, given *D*. But there is a second relationship between $\bar{w}$ and *D*, namely the advective-diffusive balance, and hence both $\bar{w}$ and *D* can be determined. It turns out that in this way, we can determine not only scales for $\bar{w}$ and *D* but the complete density structure along the side boundaries and through thermal wind the complete dependence of overturning strength on latitude. The theoretical predictions underestimate the amplitude of both overturning and heat transport by about a factor of two [25], but the meridional structure of the numerical solution is reproduced reasonably well (figure 4). Although there are discrepancies, theoretically predicted overturning correctly has a maximum near but not at the northern boundary, and the meridional dependence of heat transport is very well predicted north of 20° N. Given that there were no previous theoretical predictions of how overturning and heat transport depend on latitude, the results seemed encouraging.

Note that in the presence of slanting sidewalls, the connection between density differences and the meridional overturning is considerably more complex. A rigorous analysis of reproducing GCM MOC from GCM density differences was presented 10 years later by Joël Hirschi [35], and still later a masters student, Jörn Callies, constructed a proper numerical solution of the boundary-layer picture of the THC [36]; both papers indirectly confirmed the somewhat ad-hoc approach of Marotzke [25]. A general theoretical account of connecting boundary density to the MOC in the presence of slanting sidewalls was finally presented by Hughes *et al*. [37].

## 5. From theory to experiment design…

Not long after the paper on boundary mixing and THC theory was published [25], I received the invitation from the organizing committee of the World Ocean Circulation Experiment (WOCE) conference in Halifax to stand in for Alain Colin de Verdière as the invited speaker on ‘What governs the interior flow?’ Rather immodestly, I began my presentation by juxtaposing the two well-known ways of integrating ocean circulation into two-dimensional summaries: On the one hand, we had the vertically averaged horizontal circulation, comprising most notably the wind-driven gyres and represented well in its most fundamental features by the barotropic circulation models associated with the most celebrated names in physical oceanography, Henry Stommel and Walter Munk. On the other hand, we had the zonally averaged MOC, comprising most notably the THC, but with a virtually non-existing body of theory to explain its strength and meridional or vertical structure. I said ‘virtually non-existing’ because I was to boldly claim that I could offer elements of a theory of the THC (see figures 3 and 4, from [25]).

The talk went as well as I could have hoped for, but what surprised me the most was that my former colleague from Kiel, Uwe Send, approached me afterwards and asked me whether I thought the concepts I had presented could be used to monitor the THC in the Atlantic by measuring density profiles only at the eastern and western boundaries. I responded, and I hope my account here makes clear how strong my conviction was, that my paper was meant as a purely and deeply theoretical exercise, that the thought had not crossed my mind that my concepts could be interesting to observational oceanography, but that I would think about the prospects for monitoring the THC this way.

In February 1999, Detlef Stammer and I submitted a proposal to the US National Science Foundation (NSF), ‘Atlantic Meridional Overturning Circulation: Sensitivity and Observing System Design’, in which we proposed to use thermal-wind endpoint calculations to test whether MOC monitoring was feasible *in a model*. We took (4.3) as our starting point and added investigation of the force balance of the external mode and the influence of irregular bottom topography [38]. Furthermore, we aimed at investigating the general sensitivity of the MOC to both forcing and initial conditions (see [39]). Respecting the magnitude of the task of implementing MOC monitoring in reality, we finished on the sentence—rather cautiously and prudently, we thought—‘Finally, we would like to stress that we would welcome any opportunity to test some of the ideas outlined here with observations, in collaboration with seagoing PI's. We realize, however, that chances are slim that such an opportunity will actually arise during the next three years’.

The response to our proposal was underwhelming. One anonymous reviewer wrote: ‘If the proposers are serious about contributing to a monitoring design, and the last sentence … leads me to suspect that they are not…’ (so much for caution and prudence in a proposal), with the panel and NSF concurring with the reviewer's critical stance: ‘The program felt … that a more convincing case should be made that monitoring the MOC transport across the N. Atlantic basin would provide a good indicator of climate variability’. The proposal was rejected; Detlef Stammer took up a professorship at Scripps Institution of Oceanography, while I took up the Chair in physical oceanography at the Southampton Oceanography Centre (SOC), UK.

Shortly after arriving at the UK, I submitted a similar proposal to the Natural Environment Research Council (NERC) of the UK, naming Detlef Stammer as an official collaborator, and received the good news that it would be funded. After some search, I managed to convince Joël Hirschi, a recent PhD from Thomas Stocker's group in Berne, Switzerland, to join me in Southampton. Originally I had planned to suggest to Joël an elegant and fundamental path to AMOC monitoring design, namely to use an adjoint model to compute what the AMOC depends on (cf., [39]), and to base the monitoring design on these results. But other events intervened, forcing us to learn how to run before we had mastered walking, and I told Joël to get us an AMOC monitoring design that *worked*, never mind elegance, and he better be quick.

## 6. …And to AMOC monitoring

What had caused the change in strategy? A month after I had taken up my position in Southampton, John Lawton, a population biologist from Imperial College, took over as Chief Executive of NERC. And shortly thereafter again, I received this e-mail from a senior colleague: ‘[John Lawton] asked whether anyone in the UK was working on [THC shutdown] and … shouldn't we be doing more? I said yes we were, at SOC, and … had just hired a hot-shot new Professor who was interested/expert in it … .’ My colleague's recommendation may have had an effect since Phil Newton, the responsible programme manager with NERC, asked me to lead the effort for establishing a thematic programme targeting AMOC stability.

As the chairman of a group planning a new programme, I felt compelled to present in the first meeting *one* innovative idea that would distinguish this programme from others. This distinction was within reach because AMOC variability and change had hitherto been the focus of modellers and not observational physical oceanographers – for obvious reasons, since continuously measuring integral transport is much more challenging than continuously measuring pointwise velocities. On the other hand, standard hydrographic sections provided only snapshots of the AMOC and heat transport [18,40], usually interpreted within a steady-state framework (e.g. [28]); repeating hydrographic sections frequently enough to provide a quasi-continuous estimate of the AMOC would be impractical due to financial and personnel limitations. But, I argued, if we were interested in the future and possibly predictions of the AMOC, we surely must continuously observe the very object of our desire. And there might just be a way of accomplishing that—endpoint monitoring!

It was with considerable trepidation that I presented this idea. Here I was, a young, newly hired professor, known perhaps for my theoretical work in physical oceanography and climate dynamics but with no seagoing experience since my PhD student days, and yet I proposed what would be one of the most ambitious field programmes around. I based it on a largely untested concept—a small number of hydrographic moorings near the boundaries and, as a reference, two standard hydrographic sections (note that [41] had used endpoint monitoring to observe the flow through Drake Passage, but application to part of the AMOC apparently was only being contemplated by Uwe Send). To my surprise, especially the most seasoned observational oceanographer in the room, Bob Dickson, took to the idea, a response that encouraged me to discuss it more extensively at SOC. An indispensable instrument for these discussions proved to be the coffee room on the sixth floor, where people from the University of Southampton (like me) would mingle with NERC division people like Stuart Cunningham and Harry Bryden. It was during these discussions—started as chats over morning coffee—that Stuart, Harry and I deepened the very tentative ideas about endpoint monitoring into a concrete plan, carried by Stuart's and Harry's extensive experience in seagoing and instrumentation.

Phil Newton encouraged us repeatedly to base a proposal for a programme not on preconceived notions of possible funding levels but on the strength of the scientific innovation. In time, the plan to use endpoint monitoring to observe the AMOC gained traction, and I received a number of invitations to present the idea. At this stage, however, we did not have very strong evidence that our strategy would actually work. Our earlier paper [39] had shown that the relationship (4.3) between the second vertical derivative of the overturning stream function and the east-west density difference across the Atlantic had some validity in our model, but the computation was very simple-minded and the residual error rather large. As one consequence, there was some opposition against the endpoint monitoring idea, even after NERC decided to fund the programme that was later named RAPID. So within RAPID, the fate of AMOC monitoring was uncertain.

**Figure 5.**
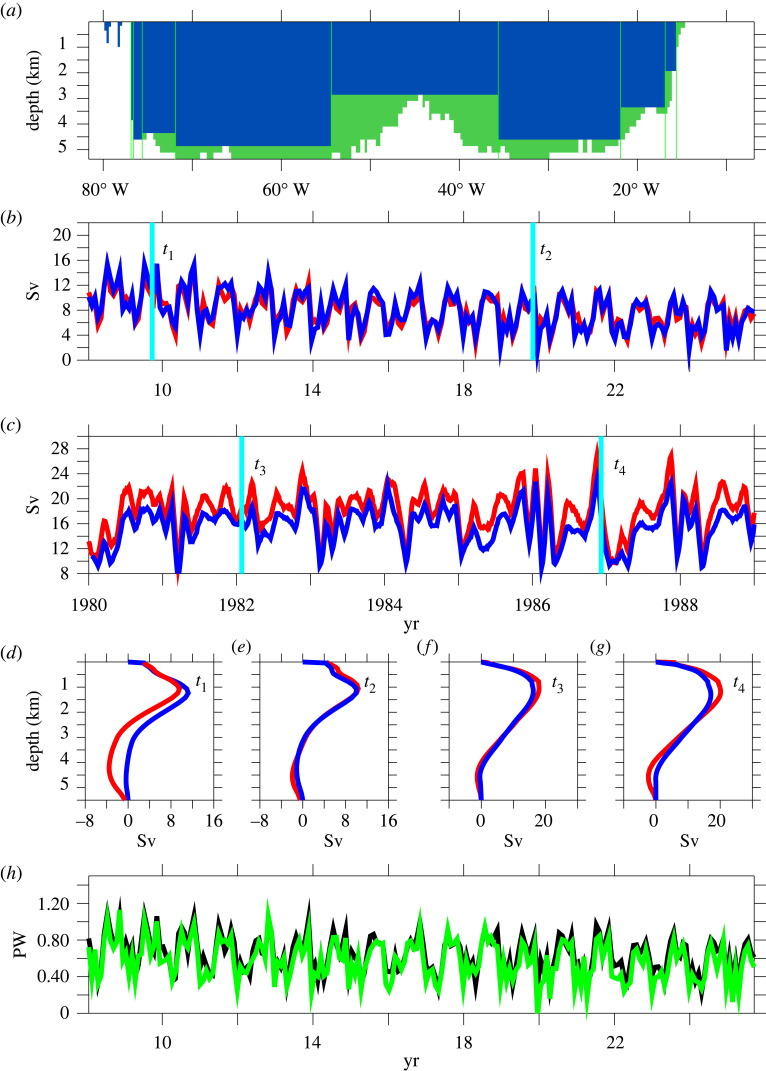
Transport reconstructions in the two OGCMs OCCAM and FLAME, based on nine vertical profiles of density and on Ekman contributions. (*a*) Distribution of vertical density profiles in OCCAM (vertical green lines). The blue shading indicates where the vertical profiles provide an estimate of the velocity shear. In the green areas (bottom triangles) the vertical shear cannot be estimated from adjacent profiles. (*b*) and (*c*) Time series for maximum AMOC based on the full model velocities (red) and reconstructed maximum AMOC (blue). Units are Sverdrups (Sv, 1 Sv ≡ 10^6^ m^3^ s^−1^). Results are shown for OCCAM (18 years, panel *b*) and FLAME (10 years, panel c). (*d*–*g*) Vertical flow patterns for AMOC (red) and its reconstruction (blue). Snapshots are shown at times *t*_1_, *t*_2_, *t*_3_ and *t*_4_ for OCCAM (panels *d*, *e*) and FLAME (panels *f*, *g*). (*h*) Net meridional heat transport at 26.5° N in OCCAM (black) and reconstructed heat transport calculated from reconstructed AMOC (green). For the reconstruction, the temperature field is assumed to be known according to one ‘hydrographic section’ at the beginning and is kept unchanged afterwards. Units are petawatts (PW, 1 PW ≡ 10^15^ W). From Hirschi *et al*. [42].

This is where Joël Hirschi's work came in. Together with a masters student, Johanna Baehr, he tested in two high-resolution ocean models whether a realistic number of vertical density profiles at 26.5° N—placed strategically to enable endpoint monitoring even in the presence of irregular bottom topography—would allow us to reconstruct the AMOC both in overall strength and vertical structure, assuming in addition mass conservation as in the analysis of hydrographic sections [40] but here applied instantaneously. The result was a resounding ‘yes’ (figure 5, from [42]). The AMOC was well reconstructed from the density information in both models, the vertical structure of zonal-mean meridional transport was captured as well, and by adding infrequent hydrographic information across the entire section we could even reconstruct heat transport variability. Figure 5 probably settled the case, and our proposal to monitor the AMOC at 26.5° N in the Atlantic [43] received top marks in the AMOC monitoring funding round of RAPID.

Why did we choose 26.5° N? This is the question I was asked more frequently than any other question about AMOC monitoring. The answer involves a mixture of scientific and logistical arguments. First, from both observations (e.g. [44]) and modelling (e.g. [45]) we expected that the maximum Atlantic heat transport was located in the subtropics, and we wanted to observe the AMOC where it mattered the most for heat transport. Second, we knew from the analyses of hydrographic sections that near 25° N the AMOC and not the gyre circulation was the dominating agent of heat transport [28,40]. Third, we felt confident that in the subtropics, heat transport fluctuations arose mainly from velocity fluctuations advecting mean heat content around, rather than mean flow advecting fluctuating heat content [46]. Fourth, 26.5° N was close to the latitude where four transoceanic hydrographic sections had been taken before, in the years 1957, 1981, 1992 and 1998. Fifth, additional experiment design studies carried out by Johanna Baehr confirmed that monitoring with a relatively small number of moorings would be unlikely to work in the subpolar gyre [47]. And sixth, choosing 26.5° allowed us to build on over 20 years of monitoring the flow through the Florida Straits [48] and the boundary current east of the Bahamas [17,49], by our now-collaborators from the US, Molly Baringer and Chris Meinen from the National Oceanic and Atmospheric Administration and Bill Johns and Lisa Beal from the University of Miami. Bill and Lisa were funded through NSF who, in a remarkable change of heart, apparently had been convinced by NERC's leadership that AMOC monitoring was, after all, a worthwhile endeavour.

The monitoring array we proposed [43] was considerably more comprehensive than the idealized system tested in Hirschi *et al*. [42]; some differences were scientific in nature, some were logistical (see [50] for what was actually deployed in the end). Hirschi *et al*. [42] assumed the use of conductivity-temperature-depth (CTD) moorings near the boundaries and the Mid-Atlantic Ridge and furthermore the use of scatterometer-derived wind stress for Ekman transport. The proposed array additionally included the cable measurements of the flow through the Florida Straits (dating back to the 1980s [48]), bottom pressure recorders to obtain some estimate of external-mode flow, and current meters in the vigorous flow near the western boundary in order not to miss important transports near topography. The relatively gentle topography on the eastern continental slope was covered through a sequence of short (*ca* 500 m) CTD moorings that, if put together, covered the water column, in addition to one full-depth CTD mooring in deep water. The prime logistical addition to the Hirschi *et al*. [42] design came through the principle that even the complete loss of any single mooring would not jeopardize the project as a whole, so substantial redundancy was built into the system [43].

Proposing a large system of moorings was one thing, making it work in practice was quite another, and this task at that time lay in the capable hands of experienced seagoing oceanographers such as Stuart Cunningham, Torsten Kanzow, Darrell Rayner, Harry Bryden and Bill Johns. RAPID started with 22 moorings, about half of them full-depth, and at times there were more than 200 CTDs in the water at a time. Dealing with this logistical challenge required efficient procedures for obtaining the instruments and other hardware and for making and analysing the measurements, procedures that were designed and implemented at the SOC by Stuart Cunningham, Torsten Kanzow and Darren Rayner. Early on some re-design of the eastern sub-array was required because of heavy and unsustainable mooring losses, presumably caused by fisheries in the area; the number of full-depth moorings was reduced and more emphasis placed on the short moorings along the continental slope [50].

Given the magnitude of the task of making all these measurements at sea, it is perhaps not surprising that it took over 3 years from the first mooring deployment to the publication of the first papers, based on the first year of data [1,3]. Cunningham *et al*. [3] demonstrated surprisingly large subseasonal AMOC fluctuations. Kanzow *et al*. [1] showed that the transport variations from the five completely independent sources of data—Ekman transport from scatterometer-based wind stress, Gulf Stream transport from the cable measurements, ‘bottom triangle’ transports from the current meters, external-mode transports from the bottom pressure recorders and the baroclinic mid-ocean transport from the moored CTDs—added up coherently such that the sums of all transports were of much smaller magnitude than the individual large fluctuations, for timescales longer than 10 days. This compensation, expected from mass conservation, to us demonstrated that the array indeed measured a signal and not noise. Furthermore, this result allowed us to use overall mass conservation to determine effectively the level-of-no-motion, even in the absence of bottom-pressure measurements. Still, the signal was claimed to be entirely overwhelmed by eddy noise [51], a claim subsequently shown to be unfounded, because eddy activity is subdued close to the boundary [2] and much smaller than assumed by Wunsch [51]. The 4-year analysis [5] confirmed that the AMOC variability did not predominantly arise from the fluctuations in Ekman transport, as was perhaps expected from model analyses for the near-equatorial latitudes [46]. Instead, there was a strong contribution to AMOC variability from the baroclinic mid-ocean transport, the part monitored with the RAPID hydrographic moorings.

## 7. Where RAPID succeeded…

The original RAPID proposal covered a 5-year period, but already by the time of submission the hope had been that the array could be sustained for longer, should it prove successful. Working tirelessly behind the scenes, Meric Srokosz, the RAPID coordinator until early 2023, managed to secure funding for an ever-longer continuation of the time series, at this writing until 2025. As experience accumulated, mooring turnaround time could be expanded, saving ship and personnel time, and the number of moorings could be reduced substantially, making the array more cost-effective (B Moat 2022, personal communication).

RAPID has accomplished quite a bit more than delivering the AMOC time series at 26.5° N. Proposals to monitor the AMOC at other latitudes almost invariably took the RAPID array as their starting point, even in cases where the monitoring is much more difficult than in the North Atlantic subtropics (e.g. [47,52–56]). An exception is, of course, Uwe Send's MOVE array at 16° N [57–60], which was conceived prior to the conception of RAPID. Nevertheless, the approach in MOVE has evolved to a system closer to the western-basin part of RAPID, in going from an array measuring only below 1000 m to a full-depth array [61,62]. Furthermore, the RAPID array has provided the reference for potential alternative AMOC observing methods, such as satellite- rather than purely moorings-based approaches (e.g. [63,64]). Frajka-Williams *et al*. [62] have presented a recent review of AMOC observing systems.

RAPID has helped create a research community beyond ‘merely’ those involved in monitoring, as witnessed by this meeting but also much more generally by fertile interactions between observational and modelling activities (in part funded through follow-up programmes to RAPID). Prior to RAPID, AMOC variability and change was the realm of the modelling and palaeo communities (e.g. [65]). This changed dramatically once the early RAPID observations came in, motivating dynamical analyses that connected hydrography and circulation [2,35,51,66–68] and also comparisons against simulations (e.g. [69,70]). The scope of AMOC work linking observations, theory and modelling has been ever-expanding since. It is impossible to do justice to this burgeoning sub-field in this single brief paper focussing more on the early developments, but an exemplar of the synthesizing papers now possible is perhaps Jackson *et al*. [71], which looks at the past 40 years of AMOC evolution based on observations, models and their combination. It is hard to imagine such a confluence of lines of evidence without the RAPID reference time series.

## 8. …And where RAPID tried but has not delivered yet

The immediate RAPID outcome was a piece of purely descriptive physical oceanography—we monitored AMOC variability at 26.5° N, something not done before or elsewhere. This accomplishment was so important to me that it did not bother me that, in the early days of RAPID, we could not provide explanations of why we observed what we did. But of course we did want to find these explanations eventually. As another example of my circuitous personal research ambitions, my starting point for AMOC work in the UK lay in theory and modelling, not in observations.

When I interviewed for the Southampton professorship in late 1998, one of the external advisors to the vice-chancellor was Herbert Huppert from Cambridge University, who drilled into me: What concretely did I plan to do? Once I understood he was after very specific answers, I said: ‘To understand the AMOC response to changes in convection’, where I had two concrete ideas in mind for pursuing this goal. The goal was reflected in the proposal to NERC for what would become the RAPID Thematic Programme [72], which listed among its science objectives as two items out of eight:

‘(1) To establish a system to continuously observe strength and structure of [AMOC].’

‘(6) To … understand the processes that connect changes in ocean convection and its atmospheric forcing to the large-scale transports … ’

Item (1) was spectacularly successful; the Marotzke *et al*. [72] proposal to establish RAPID at 26.5° N was funded, and the time series became an international standard. Item (6) essentially failed; my proposal was rejected. In hindsight, it is good that it was; the models at that time were not up to the task.

However, how the AMOC responds to changed atmospheric forcing and the changes in convective activity now matters more than ever, since despite the enormous amount of work that has occurred in the meantime (briefly alluded to in §7), I call our current understanding a litany of ignorance. Testimony for this strong statement can be found in Chapters, 2, 3, 4 and 9 of the IPCC Assessment Report 6 (AR6): We think that the AMOC will weaken in the future, but we do not know by how much and for how long, nor if and how much this weakening depends on future greenhouse gas emissions (AR6 Chapters 9 and 4 [73,74]). We are quite sure that the AMOC collapsed abruptly during the last Ice Age (e.g. [75]), but we cannot tell the likelihood of collapse under global warming ([76], AR6 Chapter 9 [73]). The AMOC has been observed comprehensively at 26.5° N since 2004, but we cannot tell whether the observed change is due to natural variability or anthropogenic change (AR6 Chapter 3 [77]); there is low confidence in longer-term proxy-derived AMOC change (AR6 Chapters 9 and 2 [73,78]). Magnitude and mechanisms of AMOC internal variability are all over the place in climate models (e.g. [79,80]) and are underestimated in most recent models [81,82]; this marks a bad foundation for Detection and Attribution approaches.

What is needed to improve our understanding, I believe, is the combination of continued AMOC observations such as RAPID and its subpolar counterpart OSNAP [56] with a new generation of climate models, at much higher spatial resolution than is currently standard practice. I postulate that the grid spacing must represent the mesoscale at least in the high-latitude open ocean (resolving it on the shelves would add another level of complication [83]). And it is not enough to apply high spatial resolution to idealized or regional forced (uncoupled) ocean models: Explicit coupling to an active atmosphere is needed for a faithful representation of water-mass formation and AMOC stability, as early research showed (e.g. [84–86]), and local or regional processes must be connected with the large-scale and ultimately global flow field.

Fortunately, models that meet these requirements have just become available, such as the new ICON atmosphere-ocean model developed at the Max Planck Institute for Meteorology that has a horizontal resolution of 5 km ([87], see [88] for a similar, recent example). The ICON simulation was exploited by Gutjahr *et al*. [89] to investigate the effect of katabatic storms off Greenland on Irminger Sea water-mass formation; the simulation represented the important processes in much better agreement with the observations, compared to lower-resolution models.

Observations and new simulations will, however, not lead to increased understanding unless they are accompanied by a proper theoretical foundation, as emphasized in the recent review of theoretical AMOC work by Johnson *et al*. [90]. In this spirit, I now want to recall the two concrete, old ideas I mention at the beginning of this section. They speak to ‘How does the atmospheric forcing lead to sinking?’ and ‘How do signals propagate meridionally?’, respectively, and while quite some work has been done on them in the meantime, I think they should play a more prominent role than they currently do.

### (a) Convective mixing and the sinking branch of the AMOC [91]

All non-oceanographers and many oceanographers believe that the sinking occurs in the deep-convection regions. But this must be wrong, definitely in the open-ocean deep-convection sites in the central Labrador and Greenland Seas, as shown by Send & Marshall [92]: The rotational effects would be so strong that vortex squashing would lead to cyclonic flow around the convective patch reaching 25 m/s if the downwelling did occur in the patch. The reason for these strong rotational effects lie in the weak stratification; the deformation radius is only around 1 km, and geostrophy is powerful even at small scales.

Send & Marshall [92] left it there—but sinking must occur somewhere. I stumbled over this issue a few years later when trying to understand the results of a particularly mad idealized numerical experiment [91]: In addition to simulating the single-basin, single-hemisphere MOC under prescribed surface density in a standard configuration, I switched off convective mixing—simply accepting if static instability ensued. In stark contrast to most people's expectation, including my own, I found that the MOC did not vanish with vanishing convective mixing but became three times stronger [91]. This surprising result forces us to accept convective mixing and downwelling as strictly different phenomena, which may but need not be co-located. Convective mixing, a.k.a. water-mass formation, modifies the density and pressure fields; this sets up modified geostrophic currents, which might impinge on the boundaries and lead to sinking there, but open-ocean deep sinking is prevented by the strong rotational constraint identified by Send & Marshall [92]. It is only if convective mixing happens to occur near the boundary that downwelling might happen in the convective patch [93]. Michael Spall in a series of papers then established the crucial role that eddies play in moving buoyancy anomalies between the region of deep convective mixing and the boundary current (e.g. [94,95]; see also [96] and the review in [90]). Note that three-dimensional dynamics are essential for these processes to unfold.

Some of these concepts had already been implicit in Cecilie Mauritzen's observations-based revised circulation scheme for the Nordic Seas ([97], a paper that deplorably we failed to cite in [91]). And the AMOC monitoring in the subpolar Atlantic (OSNAP [56]) has confirmed that sinking is not co-located with deep convection in the Labrador Sea; furthermore, the OSNAP measurements suggest that Labrador Sea deep convection is too important in standard climate models [98–100]. In summary, while important individual elements of the basic workings of the AMOC sinking branch and its variability are in place, there remain open fundamental questions on the entire chain from air–sea interaction, water-mass formation, sinking, to the AMOC.

### (b) Dynamical adjustment of the AMOC through advection [101]

The classical picture of the deep circulation assumes that downwelling at high latitudes feeds the deep western boundary current, which dominates the zonally integrated flow [32]. The spin-up of this circulation occurs through Kelvin waves [33,102] or boundary waves, which are related to Kelvin waves (see appendix in [103]). By contrast, the AMOC spin-up was demonstrated unambiguously to be due to density advection in the idealized single-basin, double-hemispheric configuration of Marotzke & Klinger [101]. By manipulating the time step for density, Kelvin and boundary waves were slowed down artificially, but there was no change in the spin-up.

The advective nature of AMOC spin-up was found in other, less idealized modelling studies (e.g. [104–106]), and some role for the advection of density anomalies finds support through observations linking dynamically active deep hydrographic anomalies in the subtropics to a subpolar origin (e.g. [107,108]). However, as Buckley *et al*. [105] already noted, the modelling and observational evidence stands ‘in contrast to numerous theoretical studies that implicate Kelvin waves in the southward communication of AMOC variability’ (page 8018 – not surprisingly, as I would add, since this theoretical work was done in a reduced-gravity framework, which excludes advection). That notion is confirmed in the recent review of theoretical AMOC work by Johnson *et al*. [90], in which the potential role of advective AMOC spin-up is not discussed. On the other hand, the robustness of the advective AMOC spin-up is unclear, owing to potential model deficiencies. Hence, I think it is fair to conclude that the question of whether density anomalies in the deep western boundary current propagate by advection or as a wave has been rarely asked and never settled.

## 9. The moral of the tale

The history of the RAPID AMOC observations, as outlined here, heaps irony upon irony. I did not leave MIT voluntarily; I was denied tenure and had to move, but it was only in Southampton and the UK that I was given the opportunity to develop the idea of AMOC monitoring. My WOCE presentation in Halifax appears to have helped a great deal in my securing the post in Southampton, and I know that this presentation and the building up of RAPID later supported my candidacy for a Max Planck directorship. Quite a number of other careers have been advanced by close involvement in RAPID, as witnessed by several principals assuming professorships.

Carl Wunsch had been my mentor at MIT and has been an inspiration for and the strongest supporter of my career, and yet Carl has been the most avid critic of the RAPID array [51,109]. This is all the more ironic since Carl was foundational for its inception; he pointed out the stability of the hydrography-derived AMOC, due to the ‘power of geostrophy’ [16, p. 6] – which encouraged me to deviate from other theoretical attempts at AMOC dynamics and to insist on geostrophy even in the boundary current and thus across the entire basin [25].

It was not only Carl who was skeptical of the RAPID approach. My other important mentor, my PhD advisor Jürgen Willebrand, told me point-blank: ‘If it were that simple, it would have been done it long ago’. And the most distinguished member of the RAPID planning committee at one point shouted out in despair: ‘Am I the only one who believes that this will never work?’ At that point we only had Marotzke *et al*. [39] and not yet Hirschi *et al*. [42], so his skepticism was perhaps understandable. But in all cases, and this is where the moral of the tale enters, I pushed back, despite the enormous respect I have always had for the insights and accomplishments of all three individuals.

## Data Availability

This article has no additional data.
